# Topographic Heterogeneity Outweighs Climate in Shaping *Artemisia* L. Species Richness and Endemism in the Hengduan Mountains, Southwest China

**DOI:** 10.3390/plants14213379

**Published:** 2025-11-05

**Authors:** Chang’an Guo, Ziwei Wang, Huifu Zhuang, Dandan Wei, Weikai Bao

**Affiliations:** 1 Chengdu Institute of Biology, Chinese Academy of Sciences, Chengdu 610041, China; guoca@cib.ac.cn (C.G.);; 2 University of Chinese Academy of Sciences, Beijing 100049, China; 3 Institute of Mountain Hazards and Environment, Chinese Academy of Sciences, Chengdu 610299, China; 4 Yunnan Key Laboratory for Wild Plant Resources, Kunming Institute of Botany, Chinese Academy of Sciences, Kunming 610200, China

**Keywords:** biogeography, biodiversity hotspot, environmental factors, topography heterogeneity, species refuge

## Abstract

*Artemisia* L. (Asteraceae) is an important ecological pioneer genus and a widely used medicinal plant group. The Hengduan Mountains (HDMs), one of the most topographically complex regions in the world, support a high diversity of *Artemisia* species. Understanding the diversity patterns of *Artemisia* species in this region is essential for conserving plant resources and promoting their sustainable use. In this study, we identified the hotspots of *Artemisia* species richness and weighted endemism in the HDMs and examined how these patterns relate to topographic heterogeneity. We confirmed the distribution of 114 *Artemisia* species across the Hengduan Mountains. Our results show clear spatial variation in *Artemisia* species diversity. Distinct hotspots were found in areas such as the Minshan Mountains, Daba Mountains, Dadu River Valley, Daxue Mountains, and Mount Gongga. The top 5% richest grid cells showed high species richness and endemism, highlighting the ecological and conservation value of these regions. Environmental analysis indicates that topographic heterogeneity, especially elevation range and surface roughness, effectively predicts diversity patterns of *Artemisia* species. Regions with more complex terrain tend to support higher species richness and endemism. These findings underscore the key role of topography in shaping *Artemisia* species diversity in mountainous areas and provide a scientific basis for future ecological research and conservation planning.

## 1. Introduction

*Artemisia* (Asteraceae) is one of the most species-rich genera in the Northern Hemisphere, containing 350–550 recognized species or subspecies [1,2]. Its ecological success comes from strong physiological plasticity. Populations are found in nearly all vegetation types, except wetlands. They occur in habitats ranging from coastal dunes to Himalayan scree at 5800 m [3,4,5]. Growth forms span annual herbs to semi-shrubs, further reflecting wide ecological amplitude [2]. However, research on the geographical distribution patterns of *Artemisia* species remains scarce. Recent phylogenomic studies have shown that *Artemisia* experienced rapid radiation and repeated adaptive divergence during the mid-Cenozoic uplift of the Qinghai–Tibetan Plateau, resulting in extensive morphological differentiation and hybridization among subgenera [6]. This evolutionary plasticity highlights *Artemisia* as an ideal model for understanding how mountain topography drives plant diversity and endemism.

The vast arid and semi-arid regions of Central Asia and northwestern North America are biodiversity hotspots and evolutionary centers of *Artemisia*, hosting a wide variety of both widespread and narrowly endemic species [7,8]. Phylogenetic and biogeographic evidence indicates that *Artemisia* originated in temperate Asia and diversified during the late Miocene, with Central and East Asia serving as major centers of radiation [9]. The complex topography and climatic oscillations of southwestern China, particularly the Hengduan Mountains, provided long-term refugia that preserved genetic diversity and facilitated repeated episodes of speciation [10]. Polyploidy, hybridization, and ecological differentiation have been key drivers of this diversification, allowing *Artemisia* species to adapt to extreme and heterogeneous environments. As dominant and often pioneer taxa across highlands and drylands, *Artemisia* plays a critical ecological role in vegetation succession, soil stabilization, and biodiversity maintenance [8]. Understanding its distribution and adaptive strategies therefore provides essential insights into plant evolution, ecological resilience, and mountain biodiversity formation.

**Figure 1:**
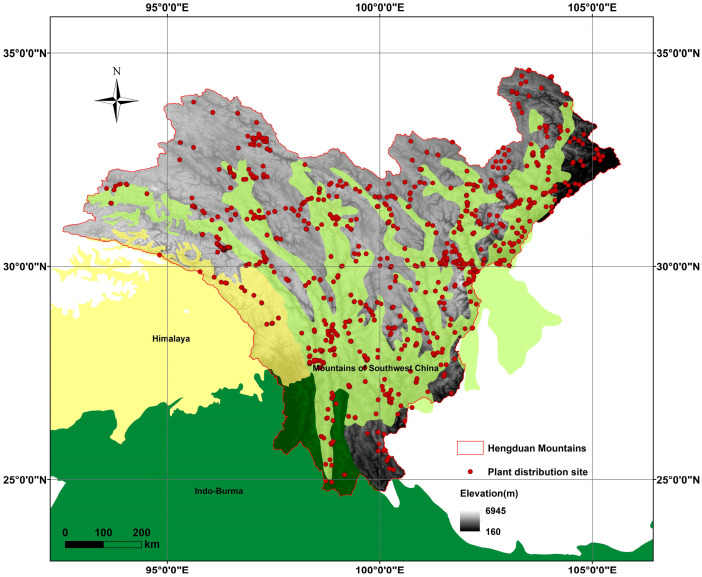
The geographical range of our study area and Distribution points of *Artemisia*. The global biodiversity hotspots involved in this region, including the Himalayas, the Indo-Burma region, and the Mountains of Southwest China.

Mountain uplift is widely recognized as a primary driver of plant diversification because steep elevational gradients compress climatic zones and create isolated “sky island” [11,12,13]. The Hengduan Mountains (HDMs) on the southeastern margin of the Qinghai–Tibet Plateau exemplify this process: six parallel ranges separated by deep gorges create local relief exceeding 3000 m within 20 km [14,15,16,17]. The Hengduan Mountains (HDMs) are situated on the southeastern edge of the Qinghai–Tibet Plateau, the world’s highest and youngest plateau. Geographically, the region spans from southwestern Gansu and southeastern Qinghai in the north, covering western Sichuan, eastern and southeastern Tibet, and extending as far south as northwestern Yunnan [18,19] (Figure 1). This region forms the core of a globally recognized biodiversity hotspot [20] and is notably rich in flora. Recent research has identified over 12,800 species of seed plants in the HDMs [19]. The HDMs is traversed by six major mountain ranges and rivers that run from north to south, unlike most other mountain ranges and rivers in China, which flow from west to east. Characterized by steep mountains, narrow gorges, and alpine plateaus, the HDMs are home to the most densely concentrated alpine gorges in the world. The average elevation of the mountain ranges exceeds 5000 m, with Mount Konka in western Sichuan standing as the highest peak at 7556 m [16,21]. The region experiences a distinct vertical climatic gradient, with mean annual temperatures ranging from 4 to 16 °C and annual precipitation between 500 and 1200 mm, decreasing sharply from the humid southeast to the arid northwest [19,21].

Plateau uplift reshaped regional climate and generated a mosaic of new habitats, promoting repeated radiations in many temperate lineages, including *Artemisia* [9,16,22,23]. Recent phylogenomic analyses confirm that *Artemisia* also experienced rapid radiation and recurrent hybridization during these tectonic and climatic upheavals, particularly across the Qinghai–Tibet Plateau and its southeastern margins [6]. The HDMs are therefore recognized as a secondary evolutionary center and migration corridor for the genus [17,24,25]. Studying the diversity patterns and underlying drivers of *Artemisia* species in the HDMs is thus of great significance for understanding its evolution and biogeography.

Topographic heterogeneity has been invoked as the primary driver of species turnover and endemism in the HDMs [16,17], but its relative importance for *Artemisia* remains unquantified. Elevation range (ER) and surface roughness (SR) capture complementary facets of terrain complexity and are known to enhance habitat diversity, micro-climatic buffering and opportunities for allopatric speciation [12]. Whether these metrics outweigh climatic, edaphic and anthropogenic factors in structuring *Artemisia* species diversity is the key unanswered question.

Previous quantitative partitioning studies have demonstrated that topographic complexity can outweigh macroclimatic factors in shaping mountain biodiversity by enhancing environmental heterogeneity and niche opportunities [26,27]. We therefore hypothesize that topographic heterogeneity is the primary environmental driver of *Artemisia* species richness and endemism in the HDMs, exerting a stronger influence than climatic, edaphic, or anthropogenic variables.

## 2. Results

### 2.1. Diversity of Artemisia Species in HDMs

Based on specimen data, there are 114 species of *Artemisia* in the HDMs. The life forms are categorized as follows: 37 (32.2%) subshrubs and 78 (67.8%) herbs, of which 68 (58.2%) are perennial herbs and 11 (9.6%) are annual herbs. Furthermore, among the 114 species of *Artemisia* found in the HDMs, 57 species (36.8%) are endemic to China (Table S1). The specimen distribution points of *Artemisia* species are mainly concentrated in the eastern regions of the HDMs and northwestern Yunnan (Figure 1). Spatial autocorrelation analysis showed no significant spatial dependence for species richness or endemism, indicating that their distributions are spatially random across the study area (Table S2).

### 2.2. Spatial Richness Patterns and Hotspots

**Figure 2:**
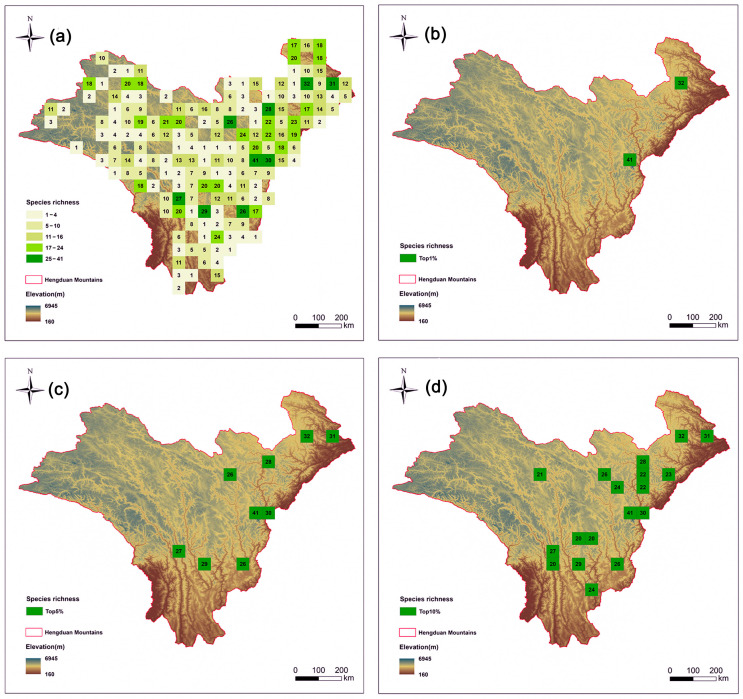
Species richness patterns of *Artemisia* species in the Hengduan Mountains. Spatial patterns of (**a**) species richness (the number is value for richness); (**b**) the top 1% of grid cells with the highest species richness of *Artemisia* species in the Hengduan Mountains; (**c**) the top 5% of grid cells with the highest species richness; and (**d**) the top 10% of grid cells with the highest species richness.

Species richness was assessed by counting the number of different *Artemisia* species present in each grid cell (Figure 2a). Taxon range sizes varied from one to 79 cells (Table S2), with species richness ranging from 0 to 41 taxa per cell. Initially, Initially, a total of 175 grid cells were ranked based on their overall species richness (Figure 2a). The top 5% hotspot regions are mainly concentrated in the eastern part of the HDMs (Figure 2c). We also compared the hotspot grids in the top 1% (Figure 2b) and top 10% (Figure 2d).

*Artemisia* species richness is notably high in two elevation ranges. The first peak occurs at low elevations (0–1000 m), corresponding to arid valley scrub, where species richness exceeds 60 species. The second peak is observed at mid–elevations (2000–4000 m), encompassing broadleaf forests, mixed conifer–broadleaf forests, and cold-temperate conifer forests, with species richness also reaching approximately 60–70 species (Figure S1).

### 2.3. Spatial Endemism Patterns and Hotspots

**Figure 3:**
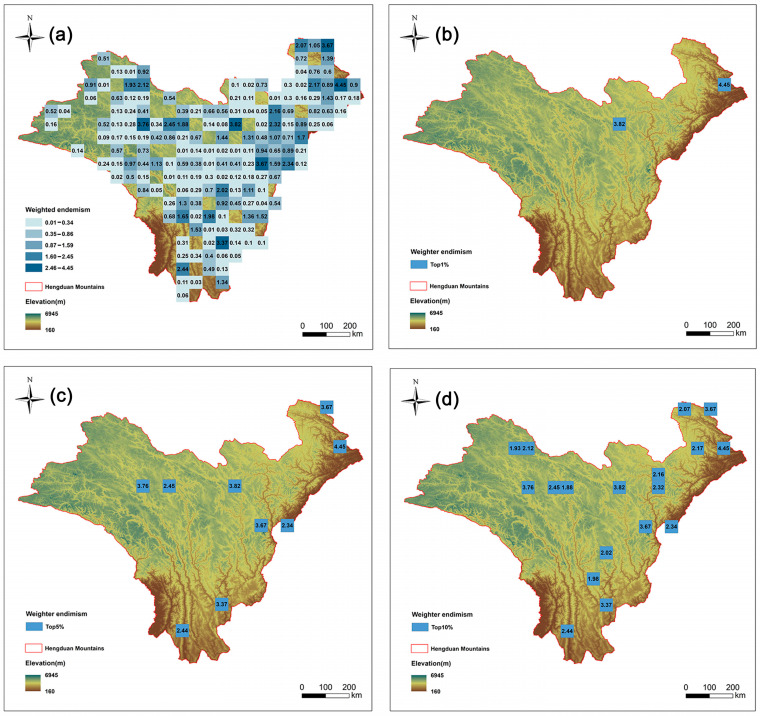
Species endemism patterns of *Artemisia* species in the Hengduan Mountains. Spatial patterns of (**a**) species endemism (the number is value for weighted endemism); (**b**) the top 1% of grid cells with the highest weighted endemism of *Artemisia* species in the Hengduan Mountains; (**c**) the top 5% of grid cells with the highest weighted endemism; and (**d**) the top 10% of grid cells with the highest weighted endemism.

This study mapped the endemism pattern of *Artemisia* species in the HDMs using a weighted average method. There are 20 species with very narrow distributions (occurring in only one grid cell) (Table S3). We further pinpointed the top nine grid cells (top 5%) with the highest endemism indices (Figure 3b). The distribution patterns of endemism and richness show a certain degree of difference. Among the nine richness grids in the top 5%, only three grids overlap with the grids in the top 5% of endemism (Table 1).

The seven identified endemism hotspots contain 66.7% of the narrowly distributed species (those occupying only one grid cell) and encompass 73.8% of all Chinese endemic *Artemisia* species in the HDMs. 

**Figure 4:**
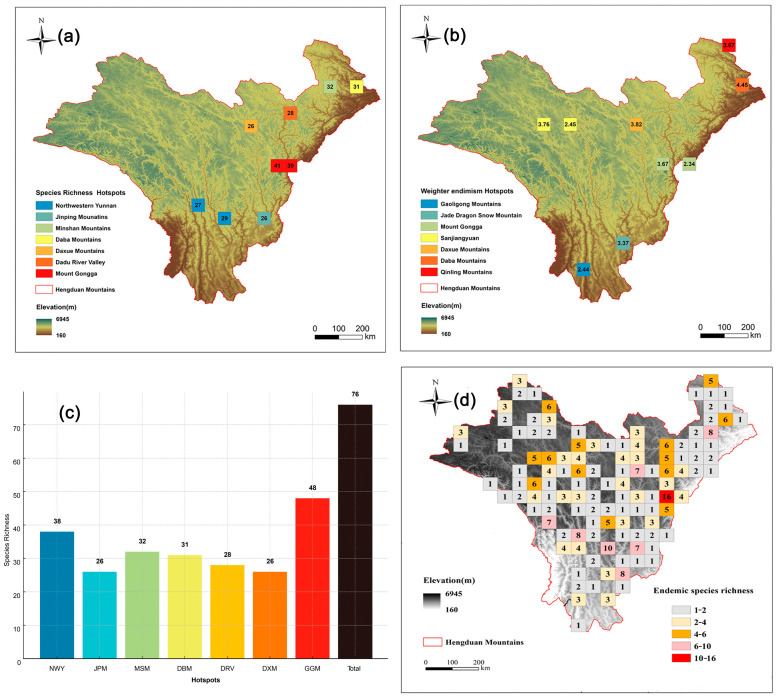
Hotspots of *Artemisia* species richness. (**a**) The nine (top 5%) highest–richness grid cells (we grouped these nine grid cells into seven geographically distinct biodiversity hotspots). (**b**) The nine (top 5%) highest–endemism grid cells. (**c**) Species richness of each hotspot region (NWY = Northwestern Yunnan; JPM = Jinping Mountains; MSM = Minshan Mounatains; DBM = Daba Mounatins; DXM = Daxue Mountains; DRV = Dadu River Valley; GGM= Mount Gongga). (**d**) Distribution pattern of endemic species richness (the numbers indicate endemic species richness).

Among these, nine cells fell within the top 5% of richness, encompassing a total of 76 *Artemisia* species (Figure 4c), which accounts for 65% of all *Artemisia* species in the HDMs. Cluster analysis using Simpson’s dissimilarity index (βsim) revealed seven hotspot regions of *Artemisia* speices richness in the HDMs (Figure 4a). The highest species diversity was observed in Mount Gongga (48 species), followed by the Northwestern Yunnan (38 species) and Minshan Mountains (32 species) (Figure 4c). We divided the top 5% of endemism grids into seven hotspot regions (Figure 4b), with the highest endemism index observed in the Daba Mountains, followed by the Daxue Mountains and Sanjiangyuan. To address the limitation of the endemism index, which overlooks widely distributed endemic species within the region, we supplemented it with a richness grid of Chinese endemic species (Figure 4d). Mount Gongga, Northwestern Yunnan, and Dadu River Valley are hotspot regions for Chinese endemic *Artemisia* species. Although our results primarily focus on the top 5% of grids based on the endemism index, alternative hotspots were also identified using the top 1% (Figure 3b) and top 10% grids (Figure 3d).

### 2.4. Topographic Heterogeneity Dominates the Variance Partitioning of Richness and Endemism

**Figure 5:**
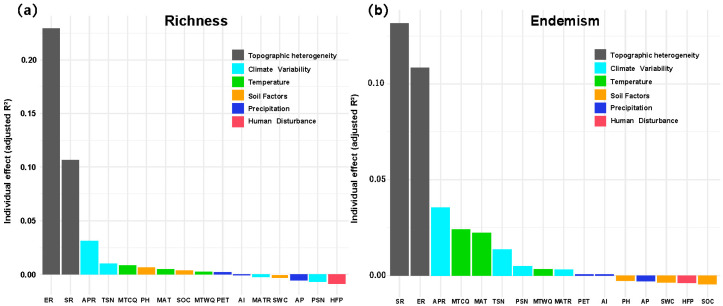
Relative importance of environmental predictors derived from GAM hierarchical partitioning. (**a**) *Artemisia* species richness and (**b**) weighted endemism across the Hengduan Mountain grid cells. Topographic heterogeneity (ER = elevation range; SR = surface roughness), climate variability (APR = annual precipitation range; TSN = temperature seasonality; PSN = precipitation seasonality; MATR = spatial range in mean annual temperature), temperature (MTCQ = mean temperature of the coldest quarter; MTWQ = mean temperature of the warmest quarter; MAT = mean annual temperature), soil factors (SOC = soil organic carbon stock; SWC = soil water capacity; pH), precipitation (AP = annual precipitation; PET = potential evapotranspiration; AI = aridity index), and human disturbance (HFP = Human Footprint Index).

Topographic metrics dominated the hierarchical partitioning output (Figure 5). For richness, elevation range alone explained 0.23 of the adjusted R^2^ and surface roughness a further 0.13, together accounting for >30% of the total unique variance. Annual precipitation range (APR, climate variability set) contributed <0.03, with all soil, temperature, precipitation and disturbance variables individually below 0.02. For weighted endemism the ranking was similar but headed by surface roughness (≈0.13) followed by elevation range (≈0.11); climate variability indices (APR, TSN, PSN, MATR) and temperature indices (MTCQ, MAT, MTWQ) provided the next 10~12%, whereas the remaining seven predictors were negligible (<0.01 each). Thus, terrain heterogeneity overwhelmingly drives both diversity metrics, with climatic variability exerting a secondary influence and all other factors making only marginal contributions.

### 2.5. Correlation of Artemisia Species Richness and Weighted Endemism with Elevational Gradients in the HDMs

**Figure 6:**
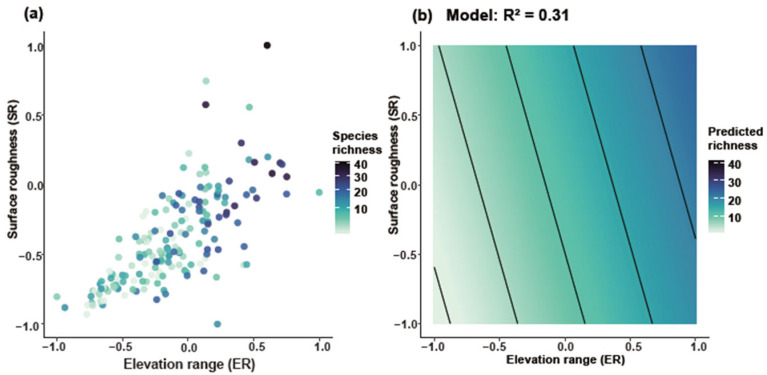

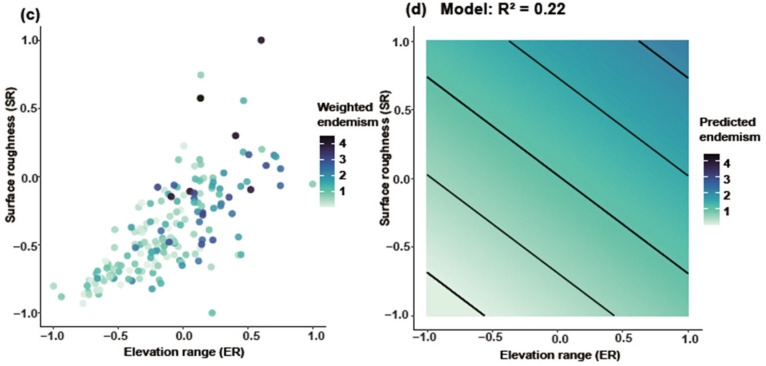
Joint effects of elevation range and surface roughness on *Artemisia* species diversity. GAM smooth (thin-plate spline, k = 4) showing the mean SR response to ER (95% CI omitted for clarity). (**a**) Relationship between ER and SR for species richness, (**b**) Predicted species richness based on the model (R^2^ = 0.31), (**c**) Relationship between ER and SR for weighted endemism, (**d**) Predicted weighted endemism based on the model (R^2^ = 0.22).

**Figure 7:**
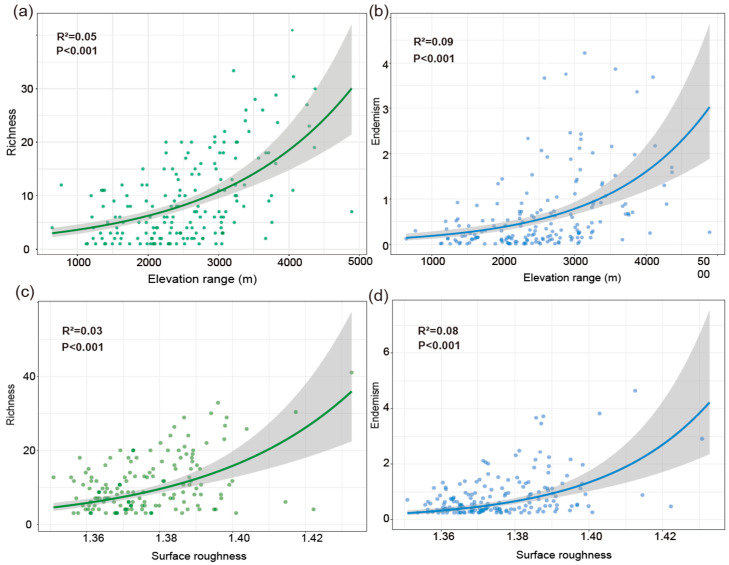
Generalized Linear Models (GLMs). (**a**) The relationship between elevation range and species richness. (**b**) The relationship between elevation range and weighted endemism. (**c**) The relationship between surface roughness and species richness. (**d**) The relationship between surface roughness and weighted endemism.

Joint response surface models based on linear regression revealed that both species richness and weighted endemism increase in tandem with elevation range (ER) and surface roughness (SR) (Figure 6). Linear regression models incorporating ER and SR as predictors explained 31% of the variation in richness (Figure 6a,b) and 22% in weighted endemism (Figure 6c,d). To further disentangle the individual effects of topographic variables, we fitted separate generalized linear models (negative binomial, log link). Species richness and weighted endemism both increased significantly with elevation range (R^2^ = 0.05 and 0.09, *p* < 0.001) (Figure 7a,b). Species richness and weighted endemism also showed significant positive relationships with surface roughness (R^2^ = 0.03 and 0.08, *p* < 0.001) (Figure 7c,d).

## 3. Discussion

### 3.1. Artemisia Species Distribution Hotspots in HDMs

We identified seven distinct hotspots of *Artemisia* species richness and endemism within the HDMs. These regions are notable for their high species richness and also harbor the majority of the endemic species in the area. Mount Gongga, in particular, stands out for its high species richness, endemism, likely due to the region’s significant altitudinal gradient that creates diverse habitats conducive to *Artemisia* species distribution [28].

The diversity hotspots of *Artemisia* species in the HDMs are predominantly located in nouthwestern Yunnan, as well as in the northern, eastern, and western regions of Sichuan. This distribution pattern parallels those observed in other plant groups, such as woody plants [29], *Rhododendron* species [30], and taxa belonging to Rosaceae family [31]. Our findings align with previous studies on vascular plants, which have identified similar hotspots in the mountains of southwestern China and the Qinghai–Tibet Plateau [32]. Our analysis underscores the significance of northwestern Yunnan and southern Gaoligong Mountain, which have been highlighted in previous research [21,33,34]. In addition, this study has revealed one new hotspot, the Dadu River Valley. The north–south orientation of the main valleys acts as an effective barrier to east–west species dispersal while facilitating north–south genetic exchange [21]. Consequently, *Artemisia* species in this region also exhibit high richenss and endemism.

The distribution pattern of *Artemisia* species reveals distinct characteristics, including increased species richness in high mountain and valley regions, rather than a pronounced concentration in the southern areas. Many northern regions also exhibit high richness, likely due to the stronger topographic heterogeneity in the north, which results in greater geographic isolation and, consequently, higher species diversity [34]. *Artemisia* species display remarkable ecological adaptability across the Hengduan Mountains, occupying habitats that range from arid river valleys to high alpine slopes. This broad ecological amplitude reflects their strong tolerance to environmental gradients such as temperature, moisture, and soil conditions, which likely contributes to their extensive distribution and diversification in the region. For instance, *A. annua* L. and *A. hedinii* Ostenf. often occur in disturbed or open habitats such as riverbanks and gravel slopes, showing rapid colonization ability and high reproductive potential [29]. In contrast, species such as *A. vestita* Wall. Ex Besser and *A. desertorum* Spreng. are well adapted to cold and arid environments, maintaining stable populations under harsh climatic conditions [35,36]. These examples illustrate how adaptive differentiation among *Artemisia* species enables them to exploit diverse ecological niches shaped by the region’s strong topographic and climatic heterogeneity.

The identification of hotspots of *Artemisia* species richness and endemism in the HDMs highlights the region’s ecological importance. The distribution pattern reveals that high species richness is concentrated in regions with significant altitudinal gradients and diverse habitats, particularly in the northwestern Yunnan and Sichuan regions. These areas also harbor a large number of endemic species, suggesting the critical role of topographic heterogeneity and climate in shaping biodiversity [37]. The identification of these hotspots not only underscores the ecological significance of the HDMs but also aligns with the global biodiversity hotspot framework proposed by Myers et al. emphasizing their conservation priority [20]. Most *Artemisia* hotspots are located in regions with complex monsoon gradients and calcareous or alluvial soils, where strong climatic seasonality and edaphic heterogeneity create diverse microhabitats that promote speciation and endemism. Recognizing these ecological commonalities provides important insights for conservation planning, suggesting that future management efforts should focus on maintaining habitat heterogeneity and protecting elevational corridors that sustain both ecological and evolutionary processes.

### 3.2. Influence of Topography Heterogeneity on Artemisia Species Diversity and Endemism

Elevational range (ER) and surface roughness (SR) are key drivers of *Artemisia* species diversity in the HDMs. Our findings show that topographic heterogeneity, particularly ER and SR, plays a major role in shaping *Artemisia* diversity patterns in the Hengduan Mountains (HDMs). Areas with the highest ER and SR closely align with hotspots of species richness and endemism. This supports the idea that steep gradients and rugged terrain create diverse niches and promote ecological isolation [26,38,39]. Large elevational ranges bring together various climatic zones and vegetation belts in small areas. This allows species with different ecological needs to coexist and promotes speciation along vertical gradients [40,41]. Surface roughness, reflected by cliffs, ravines, and peaks, generates microhabitat variation and restricts species dispersal. These features enhance the persistence of localized endemics [12,27] (Antonell). Variance partitioning shows that ER and SR together explain over 30% of the spatial variance in *Artemisia* richness. This exceeds the explanatory power of any single climatic factor. Topography thus appears more important than contemporary climate in determining diversity, consistent with findings from other mountainous regions [27,42,43].

Topography alone cannot fully explain the observed diversity patterns. Although ER and SR are dominant drivers, the relatively low R^2^ values suggest additional influences. Climate likely interacts with terrain to shape local environments [44]. For example, factors like rain shadows, snow cover, and frost zones are shaped by terrain–climate interactions. Despite high elevation, the flat interior of the Qinghai–Tibet Plateau supports fewer plant species than the rugged HDMs or Himalayas [32]. In contrast, sheltered valleys with stable microclimates may have reduced extinction rates. These areas buffered climatic fluctuations, complementing speciation driven by geological change [45].

The north–south orientation of valleys shapes both migration and isolation. North–south valleys allow species to shift latitudinally in response to climate change, serving as important genetic corridors that facilitate dispersal and gene flow along climatic gradients [17,21]. At the same time, they restrict east–west gene flow. This dual effect fosters isolation and diversification [21,33]. As a result, some areas—such as the Dadu River Valley and the southern Gaoligong Mountains—harbor unexpectedly high diversity. A national study of Chinese amphibians found similar hidden hotspots in the mountainous southwest [46]. These patterns show how topographically complex regions, acting as “sky islands,” can maintain biodiversity through repeated isolation.

To further place our findings in a global context, similar mechanisms linking topography, climate heterogeneity, and biodiversity have been observed in other major mountain systems, such as the Andes and the Caucasus, where elevation range and terrain complexity promote speciation and endemism by creating steep environmental gradients and localized refugia [38,39]. It is also important to acknowledge that elevation range (ER) and surface roughness (SR) may partially act as proxies for unmeasured environmental variability, including microclimatic heterogeneity, soil diversity, and local hydrological processes. These factors likely interact with topography to shape the fine-scale spatial patterns of *Artemisia* richness and endemism observed in the HDMs.

The HDMs act as both a cradle and refuge of biodiversity, exemplifying the principles of the mountain geobiodiversity hypothesis [47], which emphasizes how the interaction between geological dynamics and ecological processes promotes biodiversity accumulation in mountain systems. Geological processes such as uplift, river erosion, and slope instability drive rapid allopatric speciation. New habitats created during the Tibetan–Himalayan orogeny offered ecological space for adaptive radiation [16,23]. At the same time, the region’s topographic complexity buffers climatic extremes. Sheltered valleys, leeward slopes, and karst hollows may have remained humid and stable through glacial cycles. These microrefugia allowed ancient taxa to persist [17]. Thus, the HDMs not only generate biodiversity but also protect evolutionary legacies.

## 4. Materials and Methods

### 4.1. Plants Data Collection and Assembly

Specimen records of *Artemisia* species were compiled from publicly accessible plant databases, including the Global Biodiversity Information Facility (GBIF, Available online: https://www.gbif.org, accessed on 15 July 2024), the Chinese Virtual Herbarium (CVH, http://www.cvh.ac.cn, accessed on 15 July 2024), and the National Specimen Information Infrastructure (NSII, http://www.nsii.org.cn, accessed on 15 July 2024). Additionally, field survey data from several sites were incorporated to further supplement the dataset. For specimens lacking precise coordinates, geographic information was inferred from the locality descriptions on the original labels. To ensure accuracy, only specimens with verified place names and township-level precision were retained. Place names were standardized using the county-level administrative divisions released by the Chinese Ministry of Civil Affairs in May 2021. Additionally, historical place names were updated to contemporary equivalents using tools provided by the Chinese Virtual Herbarium and the National Specimen Information Infrastructure. This standardization ensured consistency in species distribution data at the township scale, resulting in a final dataset of 3276 specimen distribution records. We removed duplicate occurrence records from the same location. and verified spatial independence using Moran’s I (*p* > 0.05) (Table S2), resulting in 3276 evenly distributed georeferenced records that reliably represent large-scale biogeographic patterns of *Artemisia* species in the HDMs.

### 4.2. Mapping Distribution Ranges

To avoid over-prediction of species ranges, we did not employ species distribution models [48,49]. In this study, to minimize discrepancies in species richness estimates due to varying sizes of county-level administrative units, we converted the HDMs map into 0.5° × 0.5° equal-area grids. We compared four spatial resolutions (0.25°, 0.5°, 0.75°, and 1°) to evaluate the trade-off between spatial precision and sampling bias. Point density analysis indicated that coarser grids (≥0.75°) tend to obscure fine-scale hotspots, whereas finer grids (≤0.25°) amplify spatial noise and data sparsity effects. The 0.5° grid was therefore selected as an optimal compromise, providing stable richness estimates while minimizing overprediction and spatial artifacts.

Species richness for each grid cell was calculated as the number of species occurring within it, selecting the top 5% as biodiversity hotspots for *Artemisia* species. Many studies have shown that selecting the top 5% of land areas with the highest richness is optimal for identifying biodiversity hotspots [50,51,52]. To ensure robustness, we also compared the top 1% and top 10% thresholds. The top 5% of grids were identified as hotspots, with comparisons to the top 1% and top 10% to evaluate differences [50].

Topographic heterogeneity was characterized using two metrics: elevation range and surface roughness. The elevation range for each grid cell was calculated as the difference between the highest and lowest elevations within the cell, using elevation data from the WorldClim database (original resolution: 30 arc-seconds). Surface roughness was quantified as the ratio of the actual surface area, derived from the digital elevation model (DEM), to the planimetric area of each grid cell. This calculation was performed using the DEM Surface Tools extension in ArcGIS 10.8. To reduce the effect of skewed distributions and improve model performance, SR values were log-transformed and standardized (z-score normalization) before inclusion in the regression analyses.

### 4.3. Calculation of Diversity and Endemism Indices

In this study, species richness was calculated by simply counting the number of distinct *Artemisia* species present within the HDMs [46]. This metric represents the total number of different species occurring in a given area, providing a basic measure of taxonomic diversity.

We assessed the degree of endemism using two complementary indices: weighted endemism (WE) and endemic species richness (ESR). Weighted endemism was determined by summing the inverses of the distribution ranges of all species within each grid, where a species’ range was defined as the number of grid cells it occupied—thus assigning greater weight to narrowly distributed taxa [53]. Endemic species richness was quantified as the number of species restricted exclusively to the study region, obtained by counting species occurring only within the HDMs.

### 4.4. Evaluation of the Impact of Environmental Factors on Diversity Indicators

Seventeen raster predictors (30″ resolution) were extracted for each 0.5° × 0.5° grid cell and assigned to six functional sets: (1) human disturbance (Human Footprint Index, Global Human Footprint Dataset 2017); (2) soil (pH, organic C, water-holding capacity; SoilGrids, 0–30 cm); (3) temperature (MAT, MTWQ, MTCQ; WorldClim v2.1); (4) precipitation (AP, AI, PET; CGIAR–CSI and WorldClim); (5) climate variability (TSN, PSN from WorldClim v2.1; MATR, APR computed as within-cell spatial range); and (6) topography (elevation range, ER, and surface roughness, SR, from SRTM DEM; SR = 3–D/planimetric area, DEM Surface Tools, ArcGIS 10.8). All variables were resampled to the grid, extracted by point sampling, min–max normalized to [0, 1]. Before modeling, we examined multicollinearity among predictors using pairwise Pearson correlations and variance inflation factors (VIFs), and highly correlated variables were excluded to ensure model stability.

We first constructed generalized additive models (GAMs) in R 4.4.3 using the mgcv package, with Richness and Endemism as response variables, and all environmental variables included as smooth terms. Subsequently, we applied the gam.hp package to decompose the models and quantify the contribution of each environmental factor to the adjusted R^2^. We interpreted the HP results as approximations of the independent contribution of each smooth term within the overall GAM framework. We used linear regression and filled contour plots to visualize the spatial patterns of the joint effects of elevation range (ER) and surface roughness (SR) on species richness and weighted endemism. To further explore the influence of individual topographic factors, we fitted separate negative binomial generalized linear models (with a log link) to test the single–predictor effects of SR and ER on *Artemisia* species richness and weighted endemism.

## 5. Conclusions

This study provides a comprehensive assessment of the distribution patterns and hotspot areas of *Artemisia* species in the Hengduan Mountains, identifying a total of 114 species. Hotspots of species richness and endemism were mainly concentrated in eastern Sichuan and northwestern Yunnan. Species richness and weighted endemism showed a significant positive correlation with topographic heterogeneity, underscoring the dominant role of terrain complexity in shaping regional biodiversity. Beyond describing spatial patterns, our findings highlight key implications for biodiversity conservation and spatial prioritization, as areas with steep environmental gradients and high endemism should be considered critical for protection under future climate change scenarios. Furthermore, incorporating phylogenetic and functional diversity frameworks into future research will help elucidate how evolutionary history and ecological strategies jointly contribute to the resilience and persistence of *Artemisia* and other alpine floras within this biodiversity hotspot.

## Figures and Tables

**Table 1:** Hotspots grids of *Artemisia* species in Hengduan Mountains.

Lon	Lat	Hotspots	Richness	Endemism
32.8628	103.786	Minshan Mountains	Top 5%	Top 10%
32.8628	104.786	Daba Mountains	Top 5%	Top 5%
32.8628	103.786	Dadu River Valley	Top 5%	Top 10%
31.3628	100.786	Daxue Mountains	Top 5%	Top 5%
29.8628	102.286	Mount Gongga	Top 5%	Top 5%
29.8628	101.786	Mount Gongga	Top 5%	
27.8628	101.286	Jinping Mountains	Top 5%	
27.8628	99.7861	Northwestern Yunnan	Top 5%	Top 10%
28.3628	98.7861	Northwestern Yunnan	Top 5%	

## Data Availability

The datasets generated and/or analyzed during the current study are available from the corresponding author on request.

## Supplementary Materials

The following supporting information can be downloaded at https://www.mdpi.com/article/10.3390/plants14213379/s1: Table S1. List of *Artemisia* L. species in the Hengduan Mountains (HDMs), including their life forms and endemism status in China. Table S2. Results of the spatial autocorrelation test using Moran’s I for the selected variables, including species richness and endemism. The Moran’s I values for all variables are close to zero, and none of the *p*-values are statistically significant (*p* > 0.05), indicating the absence of significant spatial autocorrelation or clustering in the data. Table S3. Top 20 widespread species and restricted species of *Artemisia* in the study area, based on grid number and endemism metrics. Figure S1. Species richness of *Artemisia* along the altitudinal gradient in the Hengduan Mountains. Supplementary Code S1. R_Code_Artemisia_HDMs.
