# Supplementary material for: Topographic Heterogeneity Outweighs Climate in Shaping Artemisia L. Species Richness and Endemism in the Hengduan Mountains, Southwest China
Source: Plants (Basel). 2025 Nov 5;14(21):3379. doi: 10.3390/plants14213379 (PMC12609623; doi:10.3390/plants14213379)
Supplement: Supplementary file 1 [file plants-14-03379-s001.zip › Supplementary File.pdf]

**Table S1** List of Artemisa L. species in HDMs

| Latin | Life_form | Endemic species of China |
|---|---|---|
| *Artemisia abaensis* | Perennial herbaceous | Yes |
| *Artemisia absinthium* | Perennial herbaceous | |
| *Artemisia anethifolia* | Annual herbaceous | |
| *Artemisia anethoides* | Annual herbaceous | |
| *Artemisia angustissima* | Perennial herbaceous | |
| *Artemisia annua* | Annual herbaceous | |
| *Artemisia anomala* | Perennial herbaceous | Yes |
| *Artemisia argyi* | Perennial herbaceous | |
| *Artemisia atrovirens* | Perennial herbaceous | |
| *Artemisia austriaca* | Perennial herbaceous | |
| *Artemisia blepharolepis* | Annual herbaceous | |
| *Artemisia brachyloba* | Subshrubs | |
| *Artemisia calophylla* | Subshrubs | Yes |
| *Artemisia campbellii* | Subshrubs | |
| *Artemisia campestris* | Subshrubs | |
| *Artemisia capillaris* | Subshrubs | |
| *Artemisia caruifolia* | Annual herbaceous | Yes |
| *Artemisia chingii* | Perennial herbaceous | Yes |

| | | |
|---|---|---|
| *Artemisia comaiensis* | Perennial herbaceous | Yes |
| *Artemisia conaensis* | Perennial herbaceous | Yes |
| *Artemisia dalai-lamae* | Subshrubs | Yes |
| *Artemisia demissa* | Annual herbaceous | |
| *Artemisia desertorum* | Perennial herbaceous | Yes |
| *Artemisia deversa* | Perennial herbaceous | Yes |
| *Artemisia divaricata* | Perennial herbaceous | Yes |
| *Artemisia dracunculus* | Subshrubs | Yes |
| *Artemisia dubia* | Subshrubs | |
| *Artemisia duthreuil-de-rhinsi* | Perennial herbaceous | Yes |
| *Artemisia emeiensis* | Perennial herbaceous | |
| *Artemisia eriopoda* | Perennial herbaceous | Yes |
| *Artemisia erlangshanensis* | Subshrubs | Yes |
| *Artemisia flaccida* | Perennial herbaceous | |
| *Artemisia forrestii* | Subshrubs | Yes |
| *Artemisia frigida* | Perennial herbaceous | |
| *Artemisia fulgens* | Perennial herbaceous | Yes |
| *Artemisia gansuensis* | Subshrubs | Yes |
| *Artemisia giraldii* | Subshrubs | Yes |
| *Artemisia gmelinii* | Subshrubs | |
| *Artemisia gongshanensis* | Perennial herbaceous | Yes |
| *Artemisia gyangzeensis* | Subshrubs | Yes |

| | | |
|---|---|---|
| *Artemisia gyitangensis* | Perennial herbaceous | Yes |
| *Artemisia hedinii* | Annual herbaceous | |
| *Artemisia igniaria* | Subshrubs | Yes |
| *Artemisia imponens* | Perennial herbaceous | Yes |
| *Artemisia incana* | Perennial herbaceous | |
| *Artemisia indica* | Subshrubs | |
| *Artemisia japonica* | Perennial herbaceous | |
| *Artemisia kangmarensis* | Subshrubs | Yes |
| *Artemisia lactiflora* | Perennial herbaceous | Yes |
| *Artemisia lancea* | Perennial herbaceous | |
| *Artemisia latifolia* | Perennial herbaceous | |
| *Artemisia lavandulifolia* | Perennial herbaceous | |
| *Artemisia leucophylla* | Perennial herbaceous | |
| *Artemisia lingyeouruennii* | Perennial herbaceous | Yes |
| *Artemisia mattfeldii* | Perennial herbaceous | Yes |
| *Artemisia minor* | Subshrubs | |
| *Artemisia mongolica* | Perennial herbaceous | |
| *Artemisia montana* | Perennial herbaceous | |
| *Artemisia moorcroftiana* | Subshrubs | |
| *Artemisia myriantha* | Perennial herbaceous | |
| *Artemisia nanschanica* | Perennial herbaceous | Yes |
| *Artemisia neosinensis* | Perennial herbaceous | Yes |

| | | |
|---|---|---|
| *Artemisia nujianensis* | Subshrubs | Yes |
| *Artemisia occidentalisichuanensis* | Perennial herbaceous | Yes |
| *Artemisia ordosica* | Subshrubs | Yes |
| *Artemisia orientalihengduangensis* | Perennial herbaceous | |
| *Artemisia parviflora* | Perennial herbaceous | |
| *Artemisia persica* | Subshrubs | |
| *Artemisia pewzowii* | Annual herbaceous | |
| *Artemisia phaeolepis* | Perennial herbaceous | |
| *Artemisia phyllobotrys* | Subshrubs | Yes |
| *Artemisia prattii* | Subshrubs | Yes |
| *Artemisia princeps* | Perennial herbaceous | |
| *Artemisia pubescens* | Perennial herbaceous | Yes |
| *Artemisia qinlingensis* | Perennial herbaceous | Yes |
| *Artemisia robusta* | Subshrubs | Yes |
| *Artemisia roxburghiana* | Subshrubs | |
| *Artemisia rubripes* | Perennial herbaceous | |
| *Artemisia rutifolia* | Subshrubs | |
| *Artemisia scoparia* | Perennial herbaceous | |
| *Artemisia selengensis* | Perennial herbaceous | Yes |
| *Artemisia sericea* | Subshrubs | |
| *Artemisia shangnanensis* | Annual herbaceous | Yes |
| *Artemisia sichuanensis* | Perennial herbaceous | Yes |

| | | |
|---|---|---|
| *Artemisia sieversiana* | Annual herbaceous | |
| *Artemisia simulans* | Perennial herbaceous | Yes |
| *Artemisia smithii* | Perennial herbaceous | Yes |
| *Artemisia songarica* | Subshrubs | |
| *Artemisia speciosa* | Perennial herbaceous | Yes |
| *Artemisia sphaerocephala* | Subshrubs | |
| *Artemisia stracheyi* | Perennial herbaceous | |
| *Artemisia stricta* | Annual herbaceous | |
| *Artemisia sylvatica* | Perennial herbaceous | |
| *Artemisia tainingensis* | Perennial herbaceous | Yes |
| *Artemisia tanacetifolia* | Perennial herbaceous | |
| *Artemisia tangutica* | Perennial herbaceous | Yes |
| *Artemisia thellungiana* | Perennial herbaceous | |
| *Artemisia tridactyla* | Perennial herbaceous | Yes |
| *Artemisia velutina* | Perennial herbaceous | Yes |
| *Artemisia verbenacea* | Perennial herbaceous | Yes |
| *Artemisia verlotiorum* | Perennial herbaceous | |
| *Artemisia vestita* | Subshrubs | |
| *Artemisia vexans* | Subshrubs | |
| *Artemisia viscida* | Perennial herbaceous | Yes |
| *Artemisia vulgaris* | Perennial herbaceous | Yes |
| *Artemisia waltonii* | Subshrubs | |

| | | |
|---|---|---|
| *Artemisia wellbyi* | Subshrubs | |
| *Artemisia xigazeensis* | Subshrubs | Yes |
| *Artemisia yadongensis* | Perennial herbaceous | Yes |
| *Artemisia younghusbandii* | Perennial herbaceous | Yes |
| *Artemisia youngii* | Subshrubs | Yes |
| *Artemisia yunnanensis* | Subshrubs | |
| *Artemisia zayuensis* | Perennial herbaceous | Yes |
| *Artemisia zhongdianensis* | Perennial herbaceous | Yes |

**Table S2** Spatial Autocorrelation Test. shows the results of the spatial autocorrelation test using Moran's I for the selected variables, including species richness, endemism. The Moran's I values for all variables are close to zero, and none of the p-values are statistically significant ($p > 0.05$), indicating the absence of significant spatial autocorrelation or clustering in the data. This suggests that the spatial distribution of these variables is random and does not exhibit strong spatial dependence.

| **Variable** | **Moran's I** | **P-Value** |
|---|---|---|
| Richness | 0.0596 | 0.1277 |
| Endemism | -0.0371 | 0.6829 |

**Table S3** Top 20 widespread species and restricted species

| Distribution range Top 20% | Grid number | Endemism Top 20% | Grid number |
|---|---|---|---|
| *Artemisia desertorum* | 79 | *Artemisia gongshanensis* | only 1 |
| *Artemisia vestita* | 76 | *Artemisia thellungiana* | only 1 |
| *Artemisia hedinii* | 61 | *Artemisia montana* | only 1 |
| *Artemisia roxburghiana* | 60 | *Artemisia sericea* | only 1 |
| *Artemisia moorcroftiana* | 54 | *Artemisia conaensis* | only 1 |
| *Artemisia sieversiana* | 49 | *Artemisia shangnanensis* | only 1 |
| *Artemisia indica* | 48 | *Artemisia absinthium* | only 1 |
| *Artemisia japonica* | 46 | *Artemisia anomala* | only 1 |
| *Artemisia annua* | 45 | *Artemisia yadongensis* | only 1 |
| *Artemisia gmelinii* | 44 | *Artemisia younghusbandii* | only 1 |
| *Artemisia scoparia* | 42 | *Artemisia fulgens* | only 1 |
| *Artemisia neosinensis* | 40 | *Artemisia campestris* | only 1 |
| *Artemisia imponens* | 39 | *Artemisia kangmarensis* | only 1 |
| *Artemisia stricta* | 37 | *Artemisia gansuensis* | only 1 |
| *Artemisia parviflora* | 32 | *Artemisia brachyloba* | only 1 |
| *Artemisia tangutica* | 32 | *Artemisia qinlingensis* | only 1 |
| *Artemisia myriantha* | 30 | *Artemisia persica* | only 1 |
| *Artemisia princeps* | 28 | *Artemisia minor* | only 1 |
| *Artemisia verlotiorum* | 27 | *Artemisia deversa* | only 1 |

| | | | |
|---|---|---|---|
| *Artemisia argyi* | 25 | *Artemisia songarica* | only 1 |
| *Artemisia yunnanensis* | 25 | *Artemisia dalai-lamae* | only 1 |
| *Artemisia lactiflora* | 25 | *Artemisia angustissima* | only 1 |
| *Artemisia lavandulifolia* | 25 | *Artemisia phaeolepis* | only 1 |

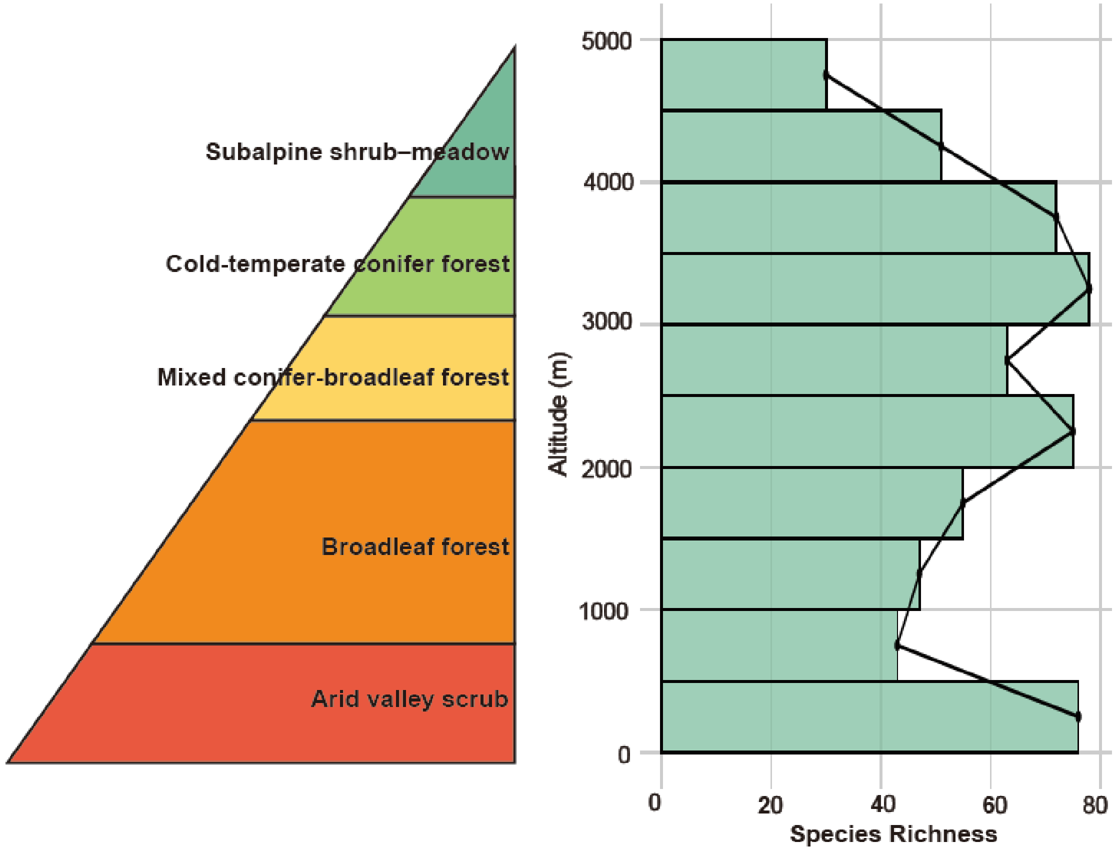


**Figure S1** Species richness along an altitudinal gradient
